# Vital protocols for PolyWare™ measurement reliability and accuracy

**DOI:** 10.3389/fsurg.2022.997848

**Published:** 2022-12-26

**Authors:** Jong Min Lee, Seung-Hoon Baek, Yeon Soo Lee

**Affiliations:** ^1^ Department of BioMedical Engineering, School of BioMedical Science, Daegu Catholic University, Gyungbuk, South Korea; ^2^ Department of Orthopedic Surgery, School of Medicine, Kyungpook National University, Kyungpook National University Hospital, Daegu, South Korea

**Keywords:** total hip arthroplasty (THA), PolyWare (PW), polyethylene wear, anteversion, lateral inclination

## Abstract

**Background and objective:**

PolyWare™ software (PW) has been exclusively used in the majority of polyethylene wear studies of total hip arthroplasty (THA). PW measurements can be significantly inaccurate and unrepeatable, depending on imaging conditions or subjective manipulation choices. In this regard, this study aims to shed light on the conditions needed to achieve the best accuracy and reliability of PW measurements.

**Methods:**

The experiment looked at how PW fluctuated based on several measurement conditions. x-ray images of in-vitro THA prostheses were acquired under a clinical x-ray scanning condition. A linear wear rate of 6.67 mm was simulated in combination with an acetabular lateral inclination of 36.6° and anteversion of 9.0°.

**Results:**

Among all the imported x-ray images, those with a resolution of 1,076 × 1,076 exhibited the best standard deviation in wear measurements as small as 0.01 mm and the lowest frequencies of blurriness. The edge detection area specified as non-square and off the femoral head center exhibited the most blurriness. The x-ray image that scans a femoral head eccentrically placed by 15 cm superior to the x-ray beam center led to a maximum acetabular anteversion measurement error of 5.3°.

**Conclusion:**

Because PW has been the only polyethylene wear measurement tool used, identifying its sources of error and devising a countermeasure are of the utmost importance. The results call for PW users to observe the following measurement protocols: (1) the original x-ray image must be a 1,076 × 1,076 square; (2) the edge detection area must be specified as a square with edge lengths of 5 times the diameter of the femoral head, centered at the femoral head center; and (3) the femoral head center or acetabular center must be positioned as close to the center line of the x-ray beam as possible when scanning.

## 1. Introduction

Wear debris-induced osteolysis and implant loosening are the primary causes limiting implant longevity after total hip arthroplasty (THA) (1, 2). Additionally, proper acetabular cup (AC) placement in THA is essential to reduce implant wear and dislocation. Thus, early detection of the complications *via* accurate measurement of wear rate and AC alignment during routine check-ups is of paramount clinical value (3–8).

Previous studies have demonstrated the high accuracy of PolyWare™ software (PW) in measuring wear rate or cup orientation (9). Even though reliable interactive computerized methods for measurements based on 2D AP x-ray images or 2D-3D registration methods have been proposed (7, 10), the majority of them have not been commercialized. In contrast, for decades PW has been the only commercially available tool to quantify THA polyethylene wear, due to its ease of use and lack of need for bead insertion or dual x-ray scanners. Because PW matches 3D sphere models representing the AC and femoral head (FH) onto the silhouettes of the AC and FH on x-ray images, it can measure the anteversion and the lateral tilt of the AC alongside polyethylene wear.

However, we found that PW measurement results can be significantly inaccurate depending on factors such as the observer's technical preferences and the features of x-ray images. Various error messages have frequently been encountered during our PW measurements due to unknown causes and PW spontaneously shutting down during measurements. The authors have categorized these errors into intrinsic and extrinsic, according to their dependency on PW performance. We believe that some errors can be reduced by optimizing the observer's choices or skill: ***Ext1***) PW's extrinsic error as a result of the original x-ray images being imported at an improper size; ***Ext2***) PW's extrinsic error as a result of the object's eccentric location away from the x-ray source-to-detector center line; ***Int1***) PW's intrinsic error, i.e., PW's functional limitation which is unable to fix the measurement error due to the asymmetrical specification of the edge detection area.

Because PW has been the only polyethylene wear measurement tool used, identifying the sources of its errors and developing a countermeasure is critical for THA research. In this regard, the current study has two aims. The first is to experimentally assess PW's extrinsic and intrinsic errors (***Ext1***, ***Ext2***, and ***Int1***). The second is to provide three technical empirical guidelines that clinicians or researchers can use.

## 2. Materials and methods

### 2.1. Study design

**Figure 1:**
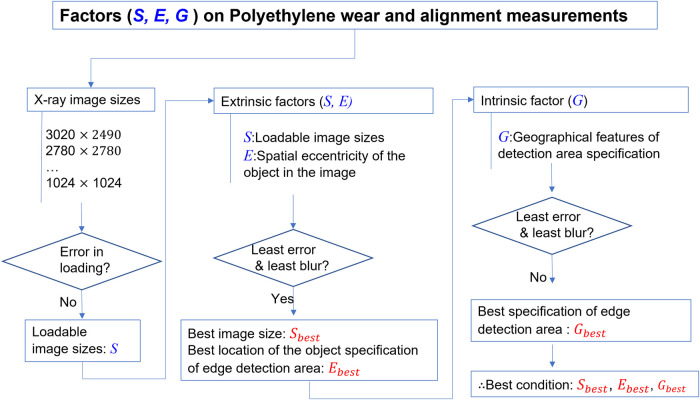
Overall process scheme of the current study.

The experiments parametrically investigated the effects of three potential error-causing factors: the size of the original x-ray image (*S*), the eccentric placement of the THA implants with respect to the x-ray source-to-detector center line (*E*), and the geometric characteristics of edge detection area definition (*G*). The *S*, *E*, and *G* factors correspond to ***Ext1***, ***Ext2***, and ***Int1***, respectively. Figure 1 shows the overall layout of the current study. To ensure the highest level of reliability for PW measurements, the three best parameters for *S*, *E*, and *G* were ultimately identified.

### 2.2. Materials

#### 2.2.1. THA prosthesis

The employed THA prosthesis set was composed of a Biolox® Delt *ϕ*28 mm femoral head (CeramTec®, Plochingen, Germany), a Trilogy® *ϕ*58 mm acetabular cup (Zimmer Biomet®, Warsaw, IN, USA), a Bencox® stem (CorenTec®, Cheon-An, Korea), and a Longevity® liner (Zimmer Biomet®, Warsaw, IN, USA). According to the authors’ experience with PolyWare measurements, the edge detection of the prostheses in x-ray images was independent of the prosthesis size. The majority of THA femoral heads have sizes between 26 and 36 mm, large enough to accurately detect the edge of the prostheses and locate the femoral head and acetabular component centers.

#### 2.2.2. Wear measurement software

**Figure 2:**
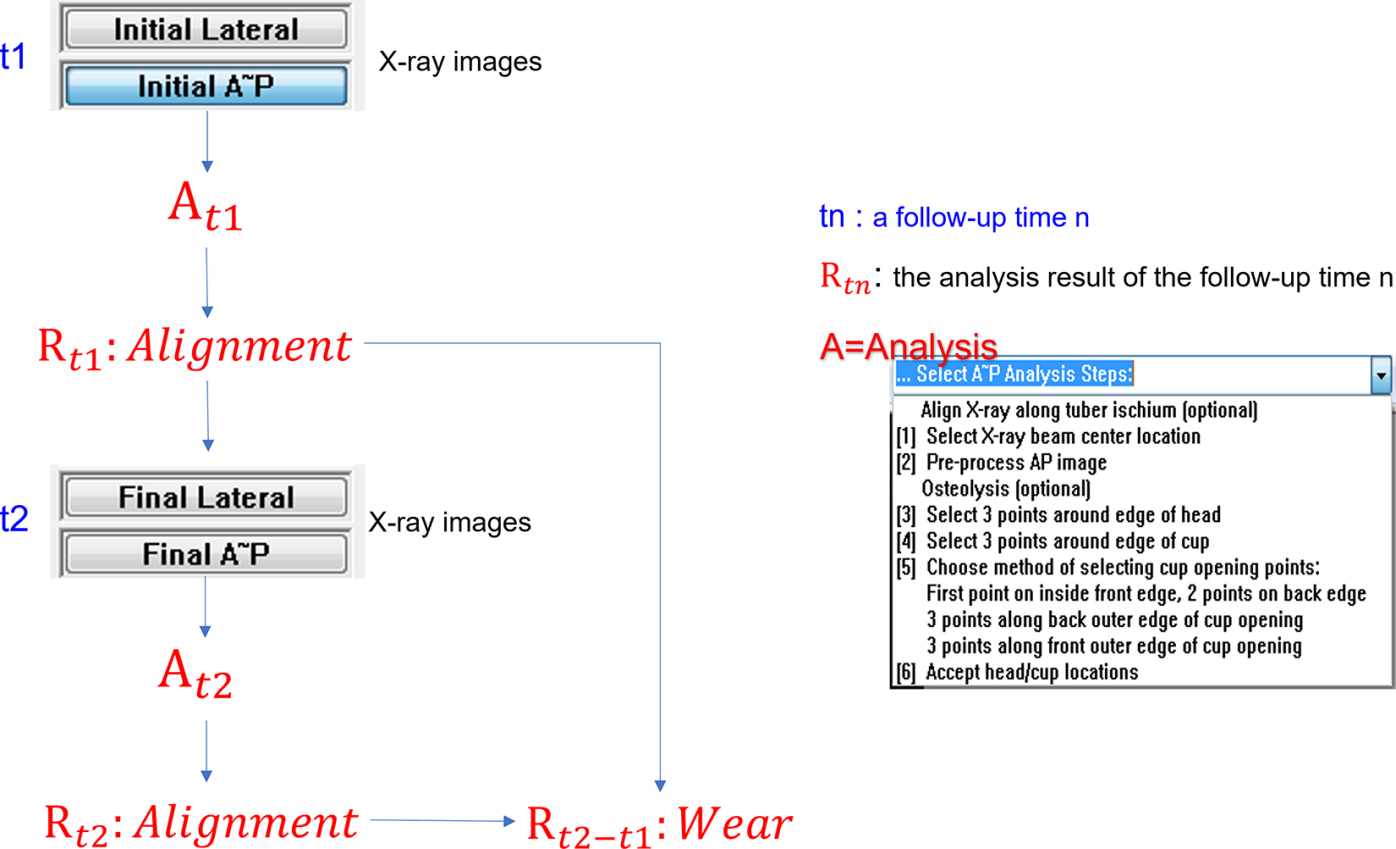
PolyWare measurement workflow.

A software called PolyWare™, v.8 (Draftware Inc., IN, USA) for radiographic measuring was used for evaluation. A PW measurement compares the analysis results of any two follow-up times. Figure 2 shows the measurement process for PW. The follow-up times can be postop (1–14 days from THA), intervals of 3 months, 6 months, 1 year, and annual increments after that. The results of the analysis include the polyethylene liner wear and the anteversion and lateral inclination of the AC. The liner wear is calculated as the difference in distance between the FH center and the AC center from the initial to the final follow-up times. The initial and final follow-up times in a PW measurement correspond to earlier and later, respectively.

#### 2.2.3. x-ray images

The images of the THA prostheses were obtained using a clinical x-ray scanner (Innovision SH, DongKang Co., Rep. Korea). The perpendicular distance from the x-ray beam source to the detector panel was fixed at 115 cm. These scanning conditions were maintained because nonuniformity in the distance or scanning direction of the beam source to the detector can lead to different results. All x-ray images were first acquired in DICOM format at a resolution of 3,020 × 3,020 pixels. They were converted to TIFF format because PW software v.8 only analyzes TIFF images or converts DICOM images into TIFF ones automatically inside the software.

#### 2.2.4. Computers

The incidence of errors in PW work may be affected by computer performance. In this regard, a laptop PC and a desktop PC with different performance levels were tested (Table 1).

**Table 1:** Specifications of the laptop and the desktop personal computers (PCs).

	Manufacturer, model	OS	RAM	CPU	Memory	Graphics
Laptop PC	Laptop PC NT270E5R, Samsung Electronics Co., Ltd., Suwon, South Korea.	Windows 7 (32bit)	8 GB	Intel Core i5 4200U	DDR 3 8 GB	Intel HD Graphics 4400, Shared memory
Desktop PC	Desktop PC, Custom-built	Windows 10 (64bit)	16 GB	Intel Core i7 4930K	DDR 3 16 GB	NVIDIA GeForce GTX 750, 1GB

#### 2.2.5. Experimental simulation setup for polyethylene wear and AC alignment

**Figure 3:**
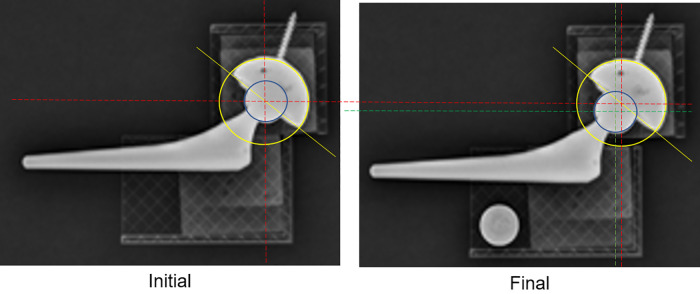
x-ray images of the initial (left) and final (right) positions, simulating cup wear by a 6.67 mm translation of the femoral stem normal to the equator plane of the AC.

Wear was replicated by translating the femoral component. The initial position of the prosthesis matched the condition in which the FH fully contacts the AC, while the final position was intended as a translation of the FH by 6.67 mm along the normal direction to the equatorial plane of the AC. x-ray images were collected before (initial) and after (final) the translation of the FH component (Figure 3). To secure the spatial link between the FH and the AC at the initial and final positions during the x-ray, alginate, an irreversible hydrocolloid, was used. Alginate powder and water were mixed in a plastic case. The mixture was left at room temperature up until the alginate started to solidify. The components of the hip prosthesis were then positioned over the alginate. The alginate foam hardened into the native shape of the prosthetic frame in 1 min.

#### 2.2.6. Measurement of true polyethylene wear and AC alignment

**Figure 4:**
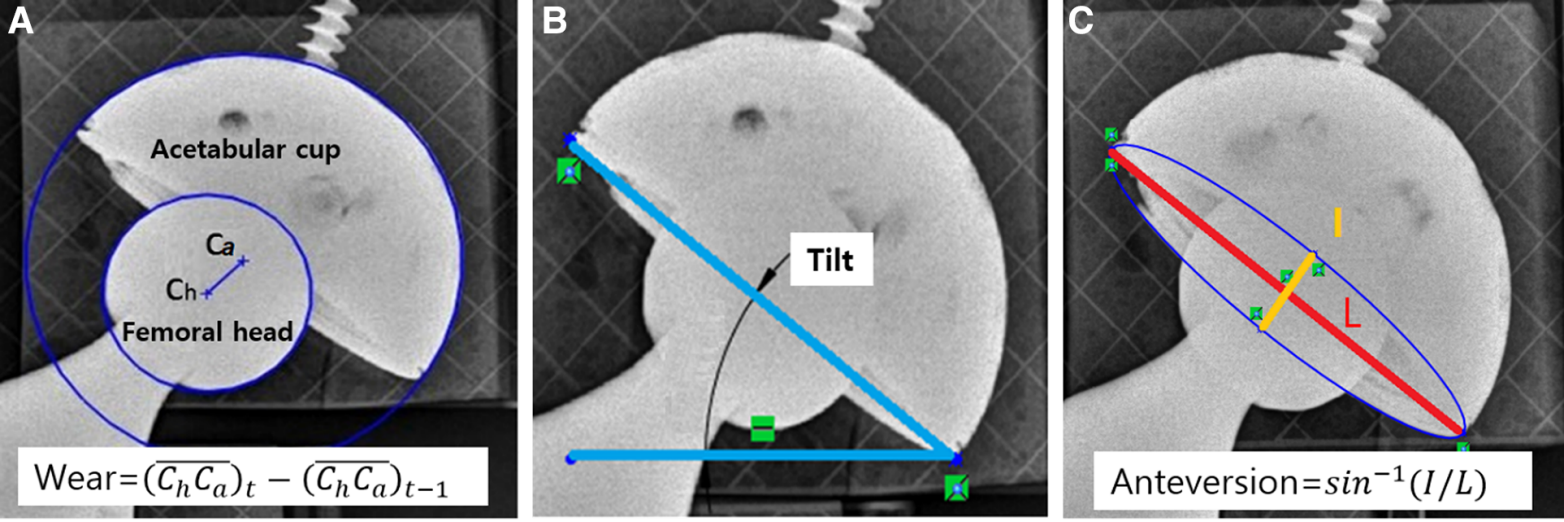
Measured values for PolyWare evaluation. (**A**) AC liner wear, (**B**) AC lateral tilt, and (**C**) AC anteversion.

A CAD measurement was used to determine the true translation of the simulated wear. The original x-ray images of resolution 3,020 × 3,020, with the FH center located at their center, are imported into CAD software, Solidworks (Dassault Systèmes, Vélizy-Villacoublay Cedex, France). The change in the intercenter distance between the femoral head and the acetabular cup was used to calculate polyethylene wear with respect to the known diameter of the FH. Additionally, the lateral tilt of the acetabular cup was calculated as the angle between the horizontal line (also known as the medial-lateral line) and the line connecting the medial-most and lateral-most points (Figure 4). AC anteversion was calculated with the Lewinnek method (11). The true translation of the FH was 6.67 mm, and the true lateral inclination and AC anteversion were 36.6° and 9.0°, respectively.

### 2.3. Compatibility of x-ray image sizes with PW

#### 2.3.1. Image loading error

When loading the x-ray images into PW, all x-ray images with a resolution of 3,020 × 3,020 or higher led to an error message. This was known as an “image loading error.” Image size is determined by several parameters, such as file format, level of color/gray expression, and resolution. Because all of the x-ray images in our study were in TIFF format with a 256 grey level, the only parameter affecting image size was resolution. Various image resolutions were tested to assess their compatibility with PW during the image loading process. The original x-ray image had a resolution of 3,020 × 3,020 and captured the FH at its center. It was subsequently shrunk to several lower-resolution images, the lowest being 1,024 × 1,024 (Table 2).

**Table 2:** Polyware compatibility tests of multiple TIFF x-ray image sizes.

Image	Resolution	1,024 × 1,024	1,076 × 1,076	1,200 × 1,200	1,300 × 1,300	1,400 × 1,400	1,500 × 1,500	1,800 × 1,800	2,494 × 2,494	2,780 × 2,780	3,020 × 3,020
	Gray bits	8	8	8	8	8	8	8	8	8	8
	Size (KB)	1,060	1,220	1,499	1,742	1,994	2,253	3,433	5.978	7,202	26,721
**Loading Error** ratio (in the desktop PC)	0/10	**0**/10	**0**/10	0/10	0/10	0/10	8/10	10/10	10/10	10/10
**Loading Error** ratio (in the laptop PC)	0/10	**0**/10	**0**/10	0/10	0/10	0/10	8/10	10/10	10/10	10/10
**Blur ratio** in the edge detection image (identical in both PCs)	5/10	2/10	4/10	6/10	5/10	5/10	2/2	NA	NA	NA
Wear (mm) True = 6.67 of All cases	6.88 (0.50)	6.79 (0.01)	6.42 (0.42)	6.64 (0.52)	6.70 (0.14)	6.61 (0.29)	6.49 (0.66)	NA	NA	NA
Wear (mm) True = 6.67 of Non-blur cases only	6.60 (0.00)	6.79 (0.00)	6.24 (0.17)	6.27 (0.28)	6.68 (0.01)	6.46 (0.25)	NA	NA	NA	NA
Lateral tilt (^∘^) True = 36.70^∘^ of All cases	36.5 (0.8)	36.0 (0.5)	36.5 (0.5)	36.2 (0.4)	36.2 (0.4)	36.4 (0.6)	36.5 (0.9)	NA	NA	NA
Lateral tilt (^∘^) True = 36.70^∘^ of Non-blur cases only	36.3 (0.7)	36.0 (0.6)	36.6 (0.6)	36.3 (0.3)	36.0 (0.4)	36.3 (0.4)	NA	NA	NA	NA
Anteversion (^∘^) True = −9.0^∘^ of All cases	−8.7 (0.4)	−8.6 (0.7)	−8.3 (0.3)	−8.7 (0.8)	−8.8 (0.5)	−8.7 (0.7)	−8.5 (0.1)	NA	NA	NA
Anteversion (^∘^) True = −9.0^∘^ of Non-blur cases only	−8.5 (0.2)	−8.6 (0.8)	−8.5 (0.2)	−8.9 (0.9)	−9.0 (0.5)	−8.5 (0.7)	NA	NA	NA	NA

**Figure 5:**
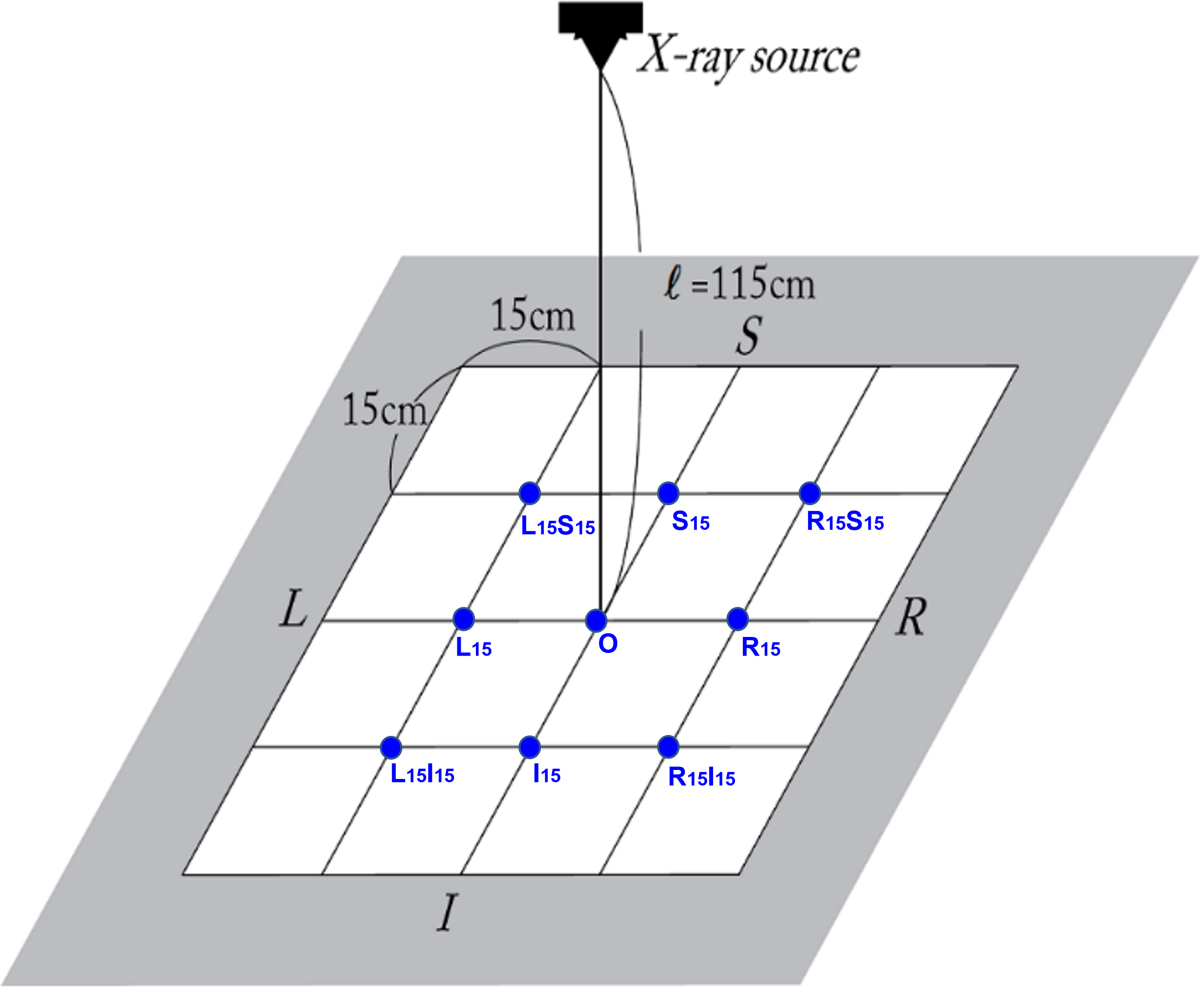
Eccentricity comparison test setup, i.e., nine spatial eccentricity modes. With respect to the center of the x-ray detector, nine spatial eccentricity locations of the THA prostheses were set up to figure out how the eccentricity of the component location affected PolyWare measurement results.

NA, not available since none of the measurement trials were successful or possible. The wear, lateral tilt, and anteversion were obtained from the only successful measurements without any blur phenomenon in both the initial and final images. These tests were performed for the x-ray image whose midpoint coincides with the center of the femoral head (*O* in Figure 5).

### 2.4. Effect of spatial eccentricity of the objects in the original x-ray images

#### 2.4.1. Test setups for spatial eccentricity modes

The distance from the x-ray beam source to an object grew as it moved away from it on a transverse plane, yet the perspective viewing angle of the object field decreased (12). As a result, the object's silhouette shape was projected differently on a detector plane, and PW measurements would give different results. We defined spatial eccentricity as the translational deviation of the FH center from the original x-ray image's middle on the same plane normal to the vector passing the x-ray source and detector centers.

Nine spatial eccentricity modes were set up *via* translating the THA prosthesis on the x-ray detector. With respect to the central location mode (*O*), other eight modes were specified *via* translating the prosthesis by 15 cm in left, right, superior, and/or inferior directions relative to the center placement mode (*O*) (Figure 5). The central mode (*O*) indicates the location of the center of the FH within the x-ray beam. All of the x-ray images used for the eccentricity tests had a resolution of 1,076 × 1,076. Without applying any rotation, the same wear of 6.67 mm was reproduced in each of the nine modes. The angular alignments of AC and acetabular liner wear should be measured at the same values because the prosthesis was only translated without rotation at all nine eccentricity modes.

### 2.5. PW compatibility of geometric features of the user-specified edge detection area

The pre-processing step termed “a pre-processing anteroposterior (AP) image” removes the superfluous region from the initially loaded AP x-ray images for measurements in a set of PW analyses. When a user assigns a rectangular area by dragging the cursor from a point to its matching diagonal point, PW magnifies the interior of the rectangle to the size of a full working window. This step only assigns the regions required for FH and AC edge detection, allowing for a more accurate, quicker analysis. Following this, PW performs edge detection for this rectangular area.

#### 2.5.1. Blurring of the edge detection area

**Figure 6:**
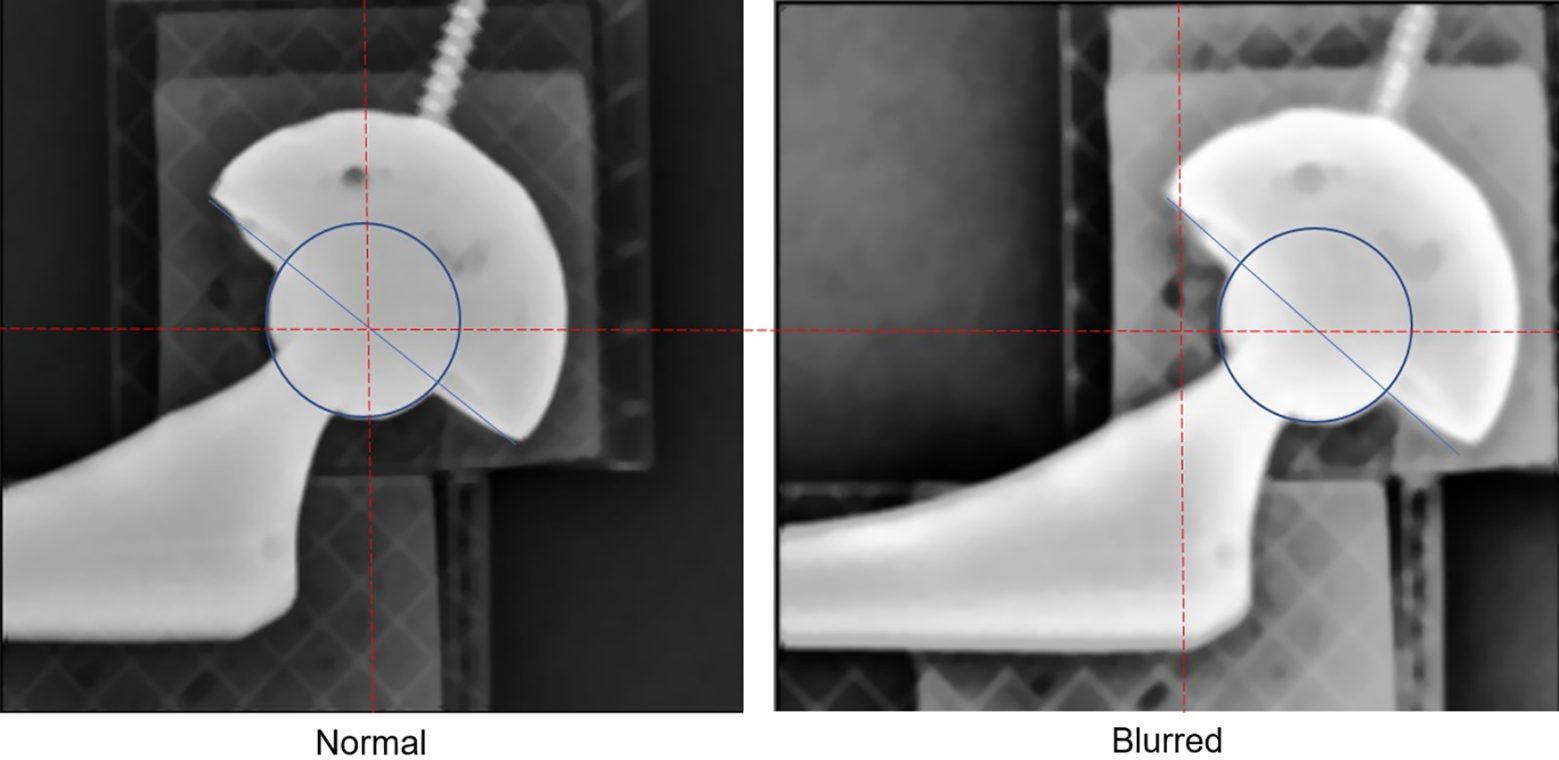
The blur of the edge detection area. For the same x-ray image, different specifications of rectangular edge detection areas result in different image sharpness. The left one is normal, but the right one is blurred. In the normal case, the rectangular edge detection area is specified such that its center is at the very center of the femoral head. In the blurred case the rectangular edge detection area is specified so that its center is considerably off the center of the femoral head, causing the edge detection area to blur.

**Figure 7:**
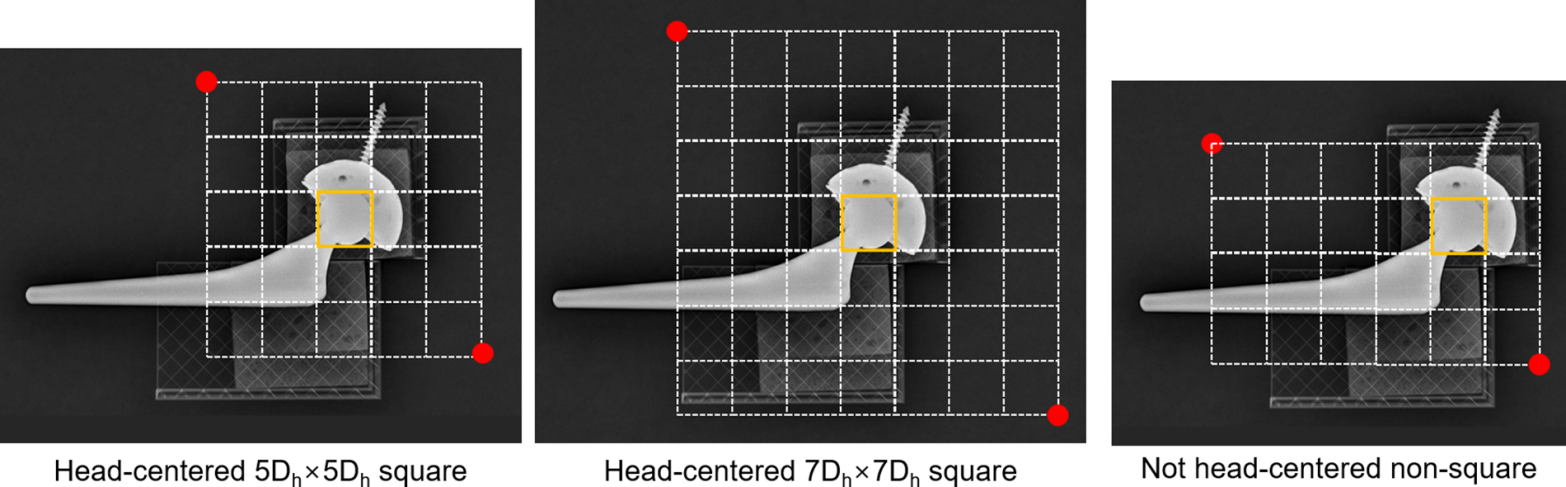
Three ways of specifying the edge detection area. The edge detection area was assigned as a rectangle whose edge lengths were 5 times (5*D_h_*) square, 7 times (7*D_h_*) square of the diameter of the femoral head component (*D_h_*), or non-square. The square areas specified were centered in the middle of the FH, whereas non-square ones were off the FH center.

Even though images were loaded into PW without any errors, PW occasionally returned a blur in the selected region during the pre-process AP step. The blur was intuitively recognizable, as in Figure 6. However, the condition in which the image blur occurs is not revealed. Standard imaging did not change the gray expression of the original x-ray image. By contrast, the blurred imaging rendered the entire edge detection area of the gray expression considerably whiter and blurrier. It was necessary to prevent the circumstances leading up to the blur. Numerous tests indicated that the placement of the user-specified edge detection area significantly affected the blurring. The frequency of the blur decreased when the center of the detection area was set as being closer to the FH center. Consequently, we hypothesized that the image blur is directly affected by the location of the FH in the edge detection area. Therefore, the following three configurations of the edge detection area were set up (Figure 7).

- Head-centered 5*D_h_* × 5*D_h_* square: the first configuration involves assigning the area as a square with edge lengths corresponding to five times the diameter of the FH (*D_h_*) and centered at the center of the FH component.
- Head-centered 7*D_h_* × 7*D_h_* square: the second configuration has the same profile as that of the first method, although its edge lengths are seven times the diameter of the FH component (*D_h_*).
- Not head-centered, non-square: the final configuration is a random specification because it is neither square-shaped nor centered at the FH center. The non-square specification indicates that the observer specifies the areas in non-squared rectangles and improvised sizes.

For this edge detection area specification test, x-ray images with a resolution of 1,076 × 1,076 were used. The image resolution of 1,076 × 1,076 was selected because it was found to be the most compatible resolution with PW (presented in the “Results” section).

## 3. Results

### 3.1. Image loading error vs. loaded image size

Concerning the image loading error, images with a resolution equal to or higher than 1,800 × 1,800 frequently failed while loading the initial or final x-ray images (Table 2). Each resolution image was tested ten times. All the images of resolutions corresponding to 2,494 × 2,494, 2,780 × 2,780, or 3,020 × 3,020 failed at being loaded into PW, i.e., the loading error rate was 10/10 = 1. The image loading error rate for 1,800 × 1,800 resolution images was 8/10. Conversely, all images with a resolution of 1,500 × 1,500 or lower were successfully loaded into PW with no errors.

In terms of occurrence rate, the image loading error was identical for the desktop PC and laptop PC (Table 2). Therefore, the PW image loading error did not depend on computer performance.

### 3.2. Blurring of the edge detection image vs. loaded image size

Only images that had been successfully loaded in PW could be used in the edge detection process. For all the images successfully loaded into PW, the edge detection area was specified in the head-centered 5*D_h_* × 5*D_h_* square.

When the edge detection area specified an error, all two successfully loaded 1,800 × 1,800 resolution images became blurry (Table 2). In contrast, images with a resolution of 1,076 × 1,076 exhibited a 2/10 blur ratio, which corresponded to the lowest blur occurrence rate among all resolutions.

### 3.3. PW-compatible geometric features of the edge detection area specification

The effects of the edge detection area's geometric feature were assessed with only the X-ray images with a resolution of 1076-1076, because all the images with this resolution were successfully loaded into PW and exhibited the least blur in the edge detection process. The incidence across 10 trials served as a measure of the blur's occurrence rate. The blur indicates that the original image is degraded by the blur created while specifying the edge detection area, and edge detection will be processed for the degraded image.

The reliability of measurements was evaluated by the incidence of blurs or unexpected errors, as shown in Table 3. When the edge detection area was specified as a square with its center in the center of the FH on x-ray images, PW measurements showed more reliability as opposed to when the center of the area was described being as randomly located off the center of the FH. The edge detection operation is terminated by an unexpected error, which indicates that the edge detection procedure returned an error message without any explanation.

**Table 3:** The measured wear for different area specifications (true wear = 6.67 mm).

	Head-centered	Not head-centered
	5D_h_ × 5D_h_ square	7D_h_ × 7D_h_ square	Non-square
Trials	Trouble	Wear (mm)	Trouble	Wear (mm)	Trouble	Wear (mm)
1	No	6.79	No	6.79	No	7.05
2	Blur	7.76	No	6.79	Error	NA
3	No	6.79	No	6.79	Blur	7.75
4	No	6.79	No	6.79	No	6.79
5	No	6.79	No	6.79	Blur	6.12
6	No	6.79	Blur	6.12	Error	NA
7	No	6.79	Blur	6.12	Blur	7.98
8	No	6.79	No	6.79	Blur	6.12
9	No	6.79	No	6.79	Error	NA
10	No	6.79	No	6.79	Blur	6.12
Total	Error: 0 Blur: 1	6.89 (0.31) of all 6.79 (0.00) of 9 N-blurs 7.76 of 1 blur	Error: 0 Blur: 2	6.66 (0.28) of all 6.79 (0.00) of 8 N-blurs 6.12 (0.00) of 2 blurs	Error: 3 Blur: 5	6.85 (0.79) of all 6.92 (0.18) of 2 N-blurs 6.82 (0.96) of 5 Blurs

NA, not available since none of the measurement trials were successful or possible. These tests were performed for the x-ray image whose midpoint coincides with the center of the femoral head (O in Figure 5). The symbol *D_h_* denotes the diameter of the femoral head component.

Ten trials of the not-head-centered, non-squared specification resulted in three unexpected errors and five blurs at the edge detection procedure. When it comes to blurring, the 7*D_h_* × 7*D_h_* square specification showed two blur incidents in ten trials, whereas the 5*D_h_* × 5*D_h_* square specification showed one blur incident in 10 trials. The wear values of both square specifications (including all the blur and non-blur situations) corresponded to 6.79 (0.00) mm, which was extremely close to the true value of 6.67 mm. In comparison, 10 trials with not-head-centered, non-squared specifications produced three unexpected errors and five blurs during the edge detection procedure. The wear of the non-head-centered random non-square specification was 6.92 (0.15) mm, which was less accurate and precise than the squared specifications.

### 3.4. Effect of the prosthesis’s eccentric placement at the time of the x-ray scanning

The eccentricity tests were performed with only the images with a resolution of 1,076 × 1,076, and their edge detection area specification was the head-centered 5*D_h_* × 5*D_h_* square. The PW measurements for each eccentricity mode were averaged from ten trials. Table 4 shows the wear amount and alignment measurement results for the nine different eccentricity modes.

**Table 4:** PolyWare measurement results for nine spatial eccentricity modes.

Eccentricity mode	Liner wear, mm True = 6.67	Lateral tilt, True = 36.7	Anteversion, True = −9.0
O	6.79 (0.00)	36.3 (0.3)	9.0 (0.6)
$L_{15}$	6.25 (0.00)	36.2 (0.6)	11.1 (0.7)
$L_{15}S_{15}$	6.52 (0.00)	36.2 (0.2)	7.9 (0.6)
$S_{15}$	6.79 (0.00)	36.7 (0.3)	4.7 (0.5)
$R_{15}S_{15}$	6.25 (0.00)	37.2 (0.3)	1.5 (0.2)
$R_{15}$	6.52 (0.00)	37.2 (0.3)	5.9 (0.6)
$R_{15}I_{15}$	6.25 (0.00)	37.2 (0.3)	9.8 (0.4)
$I_{15}$	6.00 (0.00)	37.1 (0.5)	12.6 (0.4)
$L_{15}I_{15}$	6.25 (0.00)	37.2 (0.4)	14.4 (0.3)

*L*, *R*, *S*, and *I* in the eccentricity mode represent left, right, superior, and inferior, respectively. The subscript 15 in the eccentricity mode indicates a translational distance of 15 mm.

The spatial eccentricity of the prosthesis from the original x-ray image center led to inaccurate results in wear measurement. $L_{15}$, $R_{15}S_{15}$, $R_{15}I_{15}$, and $L_{15}I_{15}$ eccentricities resulted in an error of approximately 0.42 mm, and the $I_{15}$ eccentricity resulted in an error of approximately 0.67 mm. $L_{15}S_{15}$ and $R_{15}$ resulted in an error of 0.50 mm.

AC anteversion measurements were considerably inaccurate due to any eccentricity in all directions, and, in particular, the maximum error appearing at *L*_15__I_^15^ mode by 5.4° (=14.4°–9.0°). *S*_15_ and *I*_15_ eccentricity modes resulted in anteversion measurement errors of 4.3° and −3.6°, respectively.

## 4. Discussion

In the study, we are faced with the very uncomfortable fact that some of the published PW measuring studies may not be valid if they did not acknowledge and fix the errors our research revealed. In light of our findings, we advise polyethylene wear researchers to use the following three PW measurement protocols.

### 4.1. Finding 1: Optimal size for original x-ray images ($S_{best}$)

Regarding the image loading problem, all images with a resolution of 1,500 × 1,500 or lower were successfully loaded into PW without any errors. Particularly, images with a resolution of 1,076 × 1,076 showed a two-to-ten (2/10) blur occurring ratio that was the lowest among all image resolutions. In practical situations, an original image transferred from a medical modality may be non-square (1,076 × 1,500 or 1,200 × 1,100, for example). In this instance, we recommend cropping it into a square with the original image's center at the center, changing its pixel size to 1,076 × 1,076. Therefore, an x-ray image with a resolution of 1,076 × 1,076 is optimally compatible with the PW measurement, i.e., $S_{best}$ = 1,076 × 1,076.

### 4.2. Finding 2: Optimal location of the THA prosthesis on the original x-ray images ($E_{best}$)

**Figure 8:**
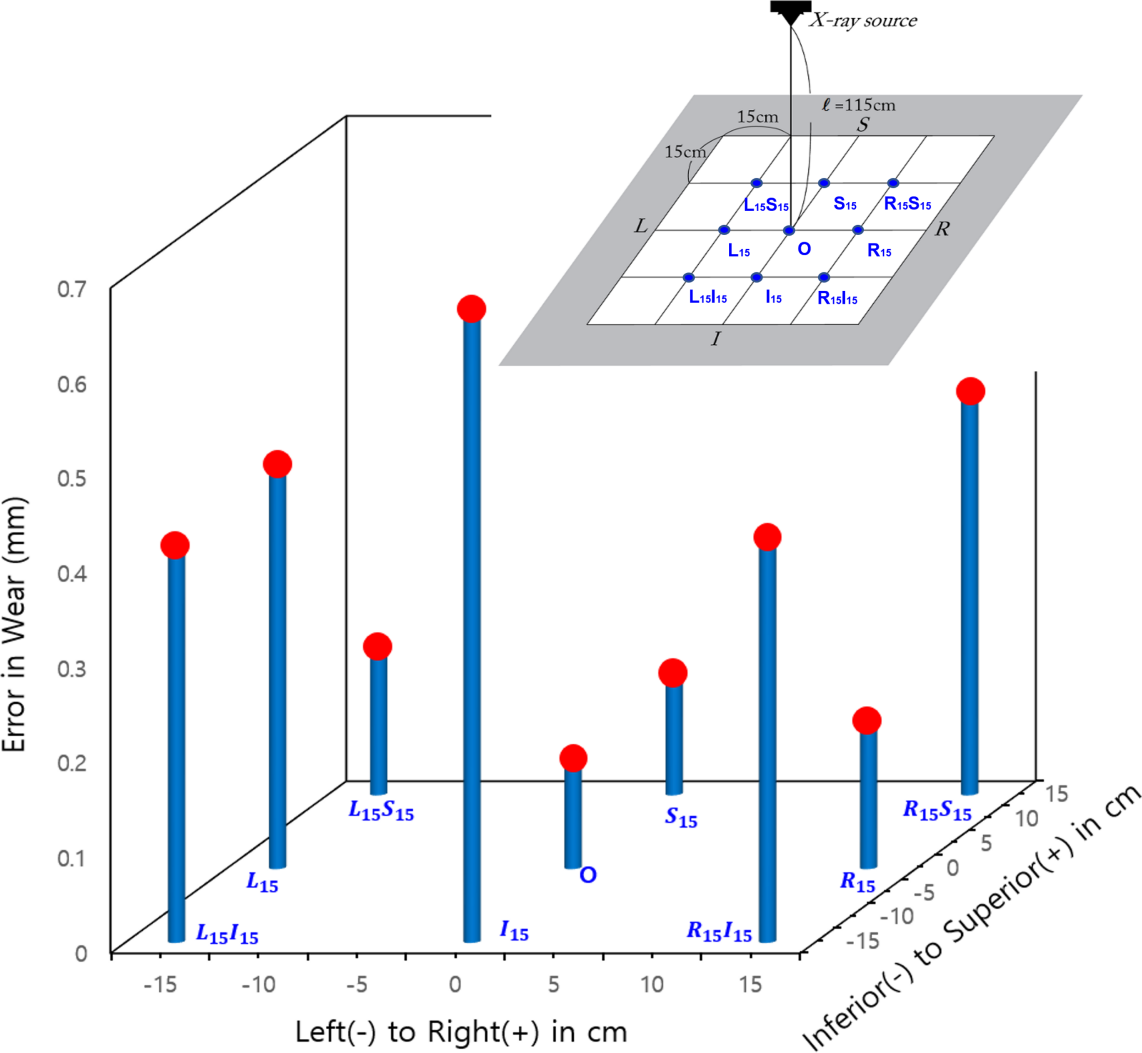
The error (in absolute values) in the wear of the femoral head's spatial eccentricity modes in the original x-rays.

**Figure 9:**
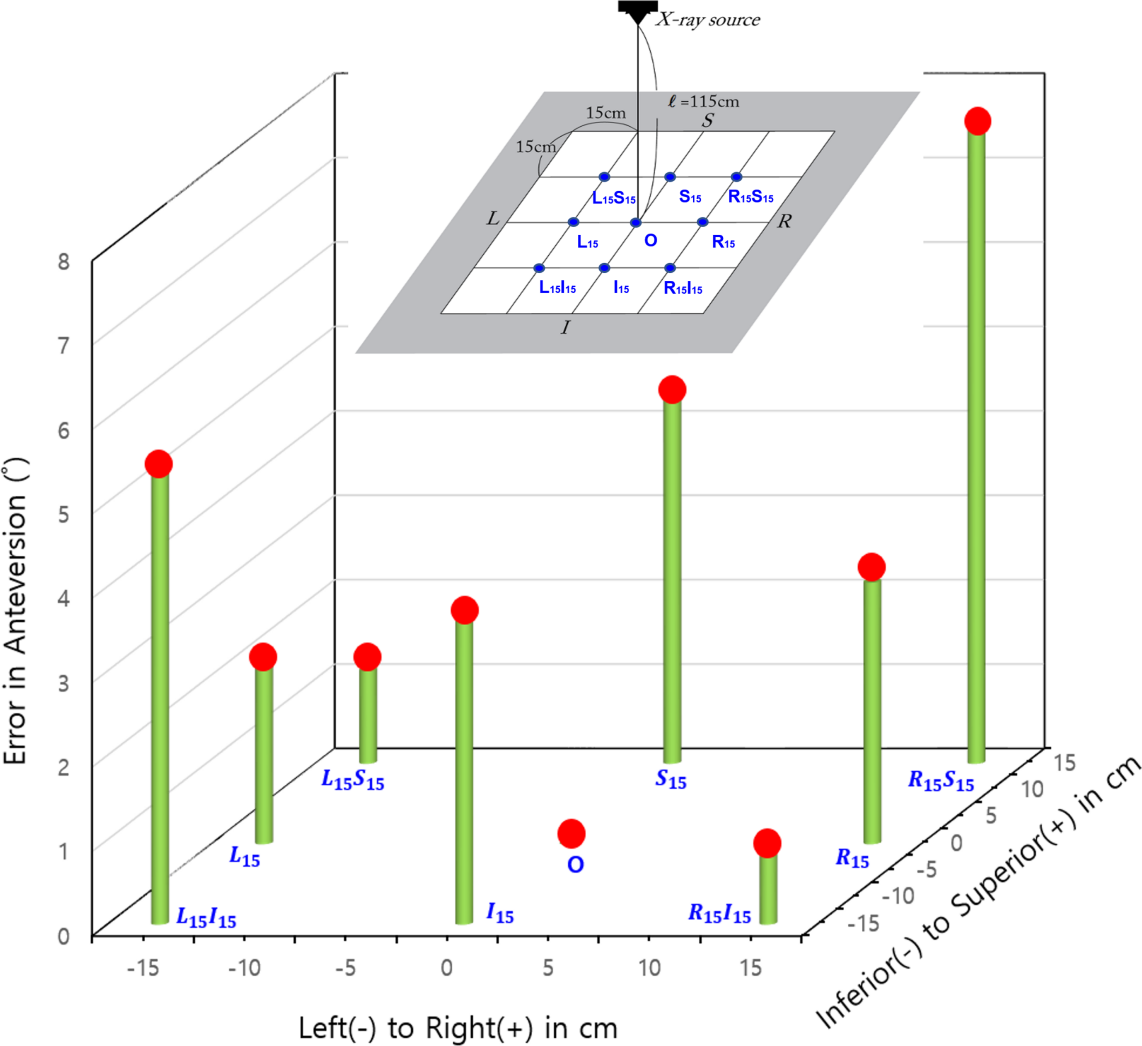
Errors (in absolute values) in the acetabular anteversion for the femoral head's spatial eccentricity modes in the original x-rays.

The eccentricity of the FH location from the x-ray beam center line significantly reduced the accuracy of the liner wear and AC anteversion measurements. The errors in Figures 8, 9 are mean deviations from the true wear and anteversion values recalculated from Table 3, respectively. It is clear that an eccentric placement of the prosthesis with respect to the x-ray beam center line leads to errors in the liner wear and AC anteversion. Because the prosthesis was placed superiorly or inferiorly in relation to the x-ray beam source, the anteversion specifically showed a greater inaccuracy. Unless the FH was placed extremely close to the central x-ray beamline at the x-ray scanning instant, the anteversion measurement by PW was unreliable.

**Figure 10:**
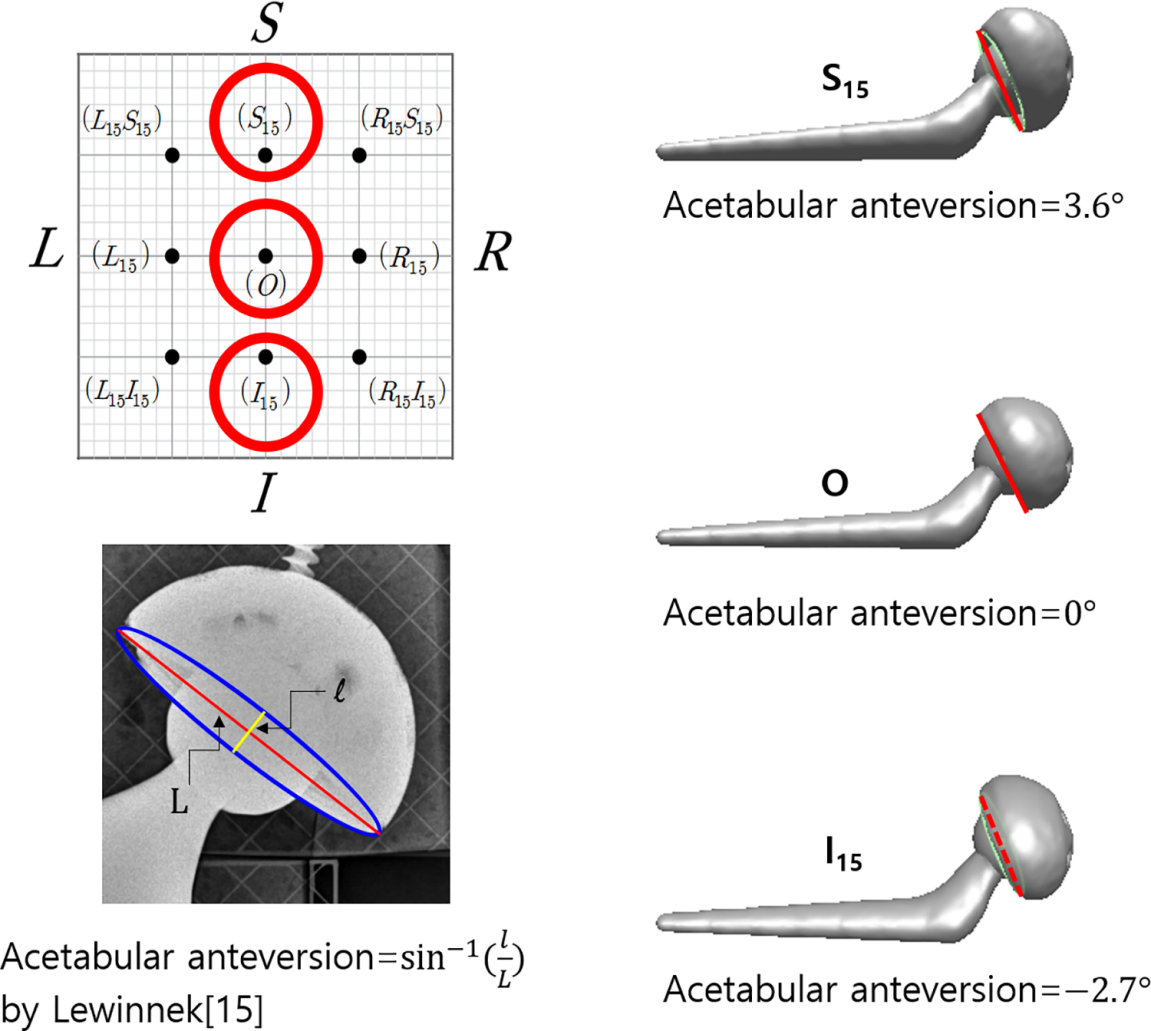
Measurement of acetabular anteversion using CAD to investigate the effect of the eccentricity of the prosthesis from the center of the x-ray beam on the acetabular anteversion. The same x-ray images used for polyethylene measurements were also used for the measurement using CAD software, i.e., Rapidform 2006^®^ (INUSTechnology, Seoul, Korea). The superior and inferior placements of the prosthesis bring about errors in acetabular anteversion by the nature of perspective x-ray imaging.

To determine why the eccentric prosthesis placement significantly affected the anteversion, we measured the anteversion of the virtual x-ray images generated by simulating a projection of the hip prosthesis 3D CAD model in a perspective view. The perspective view simulation was made with 3D CAD software, i.e., Rapidform 2006® (INUSTechnology, Seoul, Korea). With a source-to-detector distance of 394 cm, Rapidform 2006 creates a virtual perspective image in which the proximal edge of a 100cm×100cm×100cm cube is projected as 130 cm on the detector plane. Figure 10 demonstrates changes in liner wear based on superior and inferior eccentricity modes. The anteversion was calculated *via* the Lewinnek method (11). The superior and inferior 15 cm eccentricity modes were 4.3° and 3.6° of over- and under-anteversion, respectively. From our CAD measurement using Rapidform, it is postulated that PW uses the Lewinnek method to calculate acetabular anteversion. It is concluded that the acetabular measurement is only valid when the center of the FH (or similarly, the center of the AC) is placed very close to the center line of the x-ray beam. As a result, eccentricity significantly impairs the accuracy of measurements of wear and acetabular anteversion; thus, the FH center should be positioned along the center line of the x-ray beam ($E_{best}$ = O).

### 4.3. Finding 3: Optimal specification of the edge detection area ($G_{best}$)

The pre-process AP image in PW measurements required cutting out unnecessary portions from the originally loaded image. The image remaining after the pre-processing was used for edge detection of the FH and AC. The occurrence of image blur was influenced by the geometric characteristics of the region that users had specified for the pre-processing. The geometric features of the selected area include size and symmetry with respect to the center of the FH. In the current study, the asymmetry of the specified area increased the likelihood of a blur. Because the magnified process image can be more accurately analyzed for edge detection, the head-centered 5*D_h_* × 5*D_h_* square is preferable to the head-centered 7*D_h_* × 7*D*_h_ square. In this sense, we postulate that a head-centered 3*D*_h_ × 3*D*_h_ square would also be preferable. The optimal geometric specification mode of the image processing for edge detection corresponds to the head-centered 5*D_h_* × 5*D_h_* square, i.e., $G_{best}$ = head-centered 5*D_h_* × 5*D_h_* square or probably the head-centered 3*D_h_* × 3*D_h_* square.

The current research presents several limitations. First, because the study only used prostheses rather than including real tissues such as bones and soft tissues, the x-ray images used here are clinically impractical. There should be a small occlusion when tissues are absent around the prostheses; as a result, the outline of the prostheses will be more visible than when tissues are present. However, the current study aims to evaluate measurement accuracy. To assess accuracy, the true wear rate was translated into a precise simulation, and we compared the measured values to that true rate. Real clinical patient hip images cannot provide a true wear value since we are not allowed to measure the true AC wear of living individuals by surgically opening them and taking direct measurements. Additionally, the accuracy was also hindered by the difficulty of standardizing complex human tissue shapes and material compositions around THA prostheses during each x-ray scanning. Hence, in the current study, x-ray images were obtained without considering human tissues, to control accurately wear simulation by translating the femoral component. In future studies, a simulation may be developed to represent tissues around the prostheses. Second, the resolution and aspect ratio of the original x-ray images that were tested did not cover all possible variations. Clinical x-ray images may have a variety of resolutions or aspect ratios. Additionally, although PW automatically squared the imported images, practically obtained original x-ray images may not be. However, the aspect ratio will be irrelevant if the x-ray image has a resolution of 1,500 × 1,500 or lower. Thirdly, the current study investigated only one type of THA prosthesis, i.e., THA using fourth-generation ceramic-on-polyethylene articulations. When it comes to opacity in x-ray scanning, fourth-generation ceramic-on-polyethylene and metal-on-polyethylene are comparable since their liners are made of polyethylene. However, if the liners are made of radio-opaque materials like metal or fourth-generation ceramics, it can be difficult to identify the outline of the femoral head. It must be noted that PW compares patient x-ray images to measure the volume of polyethylene material worn away from the bearing surfaces of orthopedic hip implants over time (http://www.draftware.com/html/polyware.htm). Hence, PW can only be used the measure polyethylene wear.

The authors are aware of no published research that has investigated the error sources and their solutions in PW measurements. Recent literature has reported that manual measurements of the digital x-ray screen and PW measurement are comparable when it comes to measuring AC anteversion (9, 13). However, it should be highlighted that since there is no way for them to measure true polyethylene wear in living THA patients, their study only reports repeatability and not accuracy. When it comes to wear, comparing our findings with existing literature is quite limited.

## 5. Conclusion

Because PW has been the only polyethylene wear measurement tool used, identifying its sources of error and devising a countermeasure is of the utmost importance. For the best accuracy and reliability in PolyWare™ measurements, this study strongly recommends following the methodology proposed. Otherwise, the validity of the PW measurements cannot be reliably determined.

## Data Availability

The original contributions presented in the study are included in the article/Supplementary Material, further inquiries can be directed to the corresponding author.
